# TRENDS IN THE PREVALENCE OF CARDIOMETABOLIC COMORBIDITIES AMONG INDIVIDUALS WITH COGNITIVE IMPAIRMENTS

**DOI:** 10.1093/geroni/igae098.3327

**Published:** 2024-12-31

**Authors:** Yaguang Zheng, Xiang Qi, Bei Wu

**Affiliations:** New York University, New York, New York, United States; New York University, New York, New York, United States; New York University, New York, New York, United States

## Abstract

Individuals with cognitive impairment have common cardiometabolic comorbidities; however, their prevalence trends remain unknown. We aimed to address this knowledge gap by selecting participants with cognitive impairment from the Health and Retirement Study. Cognitive status was evaluated using the validated Telephone Interview for Cognitive Status (TICS), categorizing participants into normal cognition (TICS≥12), mild cognitive impairment (MCI) (TICS 7-12), and dementia (TICS < 7). The study analyzed data from 40,170 participants with cognitive impairment, with an average age of 73.4±11.2 years. Between 1996 and 2018, there was an observed increase in the prevalence of hypertension, diabetes, and stroke among adults with dementia and MCI. Specifically, hypertension prevalence rose from 60.5% to 77.0% in the dementia group and from 55.7% to 72.9% in the MCI group, diabetes prevalence increased from 21.8% to 37.9% among those with dementia and from 19.1% to 34.5% among those with MCI, and stroke prevalence escalated from 13.5% to 18.0% in the dementia group and from 8.7% to 12.3% in the MCI group. Conversely, heart disease prevalence decreased in individuals with dementia (from 35.3% to 32.2%) but slightly increased in those with MCI (from 28.4% to 29.6%). Our study found that adults with dementia or MCI had an increased prevalence trend in hypertension, diabetes, and stroke over the past two decades, although those with dementia had a decreased prevalence trend in heart disease. Further action is needed to reduce the cardiometabolic burden in this vulnerable population.

